# eTest: a limited-interaction, longitudinal randomized controlled trial of a mobile health platform that enables real-time phone counseling after HIV self-testing among high-risk men who have sex with men

**DOI:** 10.1186/s13063-020-04554-1

**Published:** 2020-07-16

**Authors:** Tyler B. Wray, Philip A. Chan, Jeffrey D. Klausner, Leandro A. Mena, James B. Brock, Erik M. Simpanen, Lori M. Ward, Stafylis Chrysovalantis

**Affiliations:** 1 Department of Behavioral and Social Sciences, Brown University School of Public Health, Providence, RI USA; 2 Division of Infectious Diseases, Warren Alpert Medical School of Brown University, Providence, RI USA; 3 Division of Infectious Diseases, Department of Medicine, David Geffen School of Medicine, University of California – Los Angeles, Los Angeles, CA USA; 4 Department of Population Health Sciences, John D. Bower School of Population Health, University of Mississippi Medical Center, Jackson, MS USA; 5 Division of Infectious Diseases, Department of Internal Medicine, School of Medicine, University of Mississippi Medical Center, Jackson, MS USA

**Keywords:** HIV testing, HIV prevention, Men who have sex with men, Counseling

## Abstract

**Background:**

HIV disproportionately affects men who have sex with men (MSM) in the USA, and new infections continue to increase, particularly among African American (AA) and Hispanic/Latino (H/L) MSM. Rates of HIV testing are particularly low among AA and H/L MSM, and innovative approaches to encourage testing may help address high incidence in these men. HIV self-testing (HST) may be an important tool for increasing rates and frequency of testing. HST may be particularly well-suited for AA and H/L MSM, given that stigma and mistrust of medical care contribute to low testing rates. Despite its promise, however, many are concerned that HST does not sufficiently connect users with critical post-testing resources, such as confirmatory testing and care among those who test positive, and that these limitations may result in delayed linkage to care.

**Methods:**

We developed a mobile health platform (eTest) that monitors when HST users open their tests in real time, allowing us to provide timely, “active” follow-up counseling and referral over the phone. In this study, 900 high-risk MSM (with targets of 40% AA, 35% H/L) who have not tested in the last year will be recruited from social media and other gay-oriented websites in several major cities. Over 12 months, participants will be randomly assigned to receive (1) HST with post-test phone counseling and referral (eTest condition), (2) HST without active follow-up (standard condition), or (3) reminders to get tested for HIV at a local clinic (control) every 3 months. Primary outcomes include rates of HIV testing, receipt of additional HIV prevention services, and PrEP initiation verified by clinical medical records.

**Discussion:**

This study tests whether providing more active counseling and referral after HST encourages more regular HIV testing and engagement with other prevention services among MSM, compared to more passive approaches or clinic-based testing alone. It will also explore the cost-effectiveness and emotional/behavioral effects of these two strategies.

**Trial registration:**

ClinicalTrials.gov identifier NCT03654690. Registered on 31 August 2018.

## Administrative information

Note: the numbers in curly brackets in this protocol refer to SPIRIT checklist item numbers. The order of the items has been modified to group similar items (see http://www.equator-network.org/reporting-guidelines/spirit-2013-statement-defining-standard-protocol-items-for-clinical-trials/).

**Table:** 

Title {1}	A limited-interaction, longitudinal randomized controlled trial of a mobile health platform that enables real-time phone counselling after HIV self-testing among high-risk men who have sex with men (eTest)
Trial registration {2a and 2b}.	ClinicalTrials.gov identifier NCT03654690. All items from the WHO trial registry data set can be found within the body of the protocol.
Protocol version {3}	Version 1
Funding {4}	R01MH114891, National Institute of Mental Health, National Institutes of Health
Author details {5a}	Wray, Tyler B.^1^, Chan, Philip A.^2^, Klausner, Jeffrey D.^3^, Mena, Leandro A.^4, 5^, Brock, James B.^5^, Simpanen, Erik M.^1^, Ward, Lori M.^4^, Chrysovalantis, Stafylis^3^, Carr, Daniel J.^1^ ^1^Department of Behavioral and Social Sciences, Brown University School of Public Health, Providence, RI ^2^Division of Infectious Diseases, Warren Alpert Medical School of Brown University, Providence, RI ^3^Division of Infectious Diseases, Department of Medicine, David Geffen School of Medicine, University of California – Los Angeles, Los Angeles, CA ^4^Department of Population Health Sciences, John D. Bower School of Population Health, University of Mississippi Medical Center, Jackson, MS ^5^Division of Infectious Diseases, Department of Internal Medicine, School of Medicine, University of Mississippi Medical Center, Jackson, MS
Name and contact information for the trial sponsor {5b}	Gregory Greenwood, National Institute on Mental Health, 1-240-669-5532
Role of sponsor {5c}	Study sponsors played no role in the study design, the collection, management, analysis, or interpretation of data, writing of the report, or the decision to submit the report for publication.

## Introduction

### Background and rationale {6a}

Although overall HIV incidence in the USA has remained stable in recent years, new infections continue to increase in certain groups of men who have sex with men (MSM) [1]. In 2014, MSM accounted for 67% of all new HIV infections [2], a rate that has risen steadily in recent years [3]. New infections are especially high among African American (AA) and Hispanic/Latino (H/L) MSM. Recent surveillance data suggests that, if current incidence trends continue, 1 in 2 AA MSM and 1 in 4 H/L MSM will be diagnosed with HIV in their lifetimes [4].

One source of new HIV infections stems from those who are aware they have HIV but who are not virally suppressed. However, another major source is the estimated 20% of MSM who are infected but unaware of their status [5]. Past modeling studies have suggested that this scenario may account for up to 50% of new infections [6, 7], prompting calls to increase the access and availability of HIV testing [8]. Despite their elevated risk, fewer than 60% of MSM report having been tested in the last 12 months, and only 20% have been tested more than once in the past year [9, 10]. AA and H/L MSM are also twice as likely as White MSM to have never tested in their lifetimes [9].

Testing is a cornerstone of HIV prevention efforts, since it can facilitate early diagnosis and treatment (i.e., “test and treat”) [11]. Studies show that this approach can reduce HIV incidence when implemented broadly [12–14], in part by reducing the time between infection and diagnosis. This gap between infection and diagnosis averages 2.6 years in some areas [15]. Expanding testing is a particularly important step in reducing new infections among AA and H/L MSM, since those who are unaware of their infection may be key drivers of incidence in these groups [16, 17]. Together, these findings suggest that innovative approaches to expanding testing are needed, particularly among AA and H/L MSM.

#### HIV self-testing (HST) could overcome key barriers to testing

In July 2012, the first rapid HIV self-test (HST) was approved by the FDA (OraSure® Technologies, Bethlehem, PA). This test uses oral fluid sampling, produces results in 20 min and can be completed entirely by end users. As a compliment to clinic-based testing, HST has the potential to reach high-risk MSM who test infrequently. Past studies show that the most prominent obstacles to clinic-based testing among MSM were concerns about confidentiality and inconvenience (e.g., travel, wait times) [18, 19]. Others show that the vast majority of MSM, and especially young MSM and those who have never tested, would prefer HST and feel they would test more often with HST [18, 20–25]. Further, HST may be particularly well-suited for increasing testing among AA and H/L MSM, given that stigma and distrust of traditional medical services are key obstacles to clinic-based testing for these men [26–28]. These findings underscore HST’s potential for overcoming barriers to testing and for encouraging those who test infrequently to do so more often. Using HST to encourage more frequent, regular HIV testing could facilitate earlier diagnosis and linkage to care, thereby improving disease outcomes [29] and reducing onward transmission [30]. For these reasons, the World Health Organization has recently recommended HST for high-risk populations and suggested that it may be key to reaching its target of diagnosing 90% of those who have HIV [31].

One strategy for increasing HIV testing among high-risk MSM involves providing free HST through the apps/sites they already use. Our past studies [32–34] show that using these apps to inform users about HST, conduct a brief risk assessment, and send an HST through the mail is acceptable and feasible. Moreover, Elliot et al. [35] also demonstrated that sending HST to app users successfully detected new HIV infections, with 77% of new diagnoses made at CD4 counts > 350 cells/μL, suggesting that HST might facilitate early diagnosis. Together, this work shows that providing HST to high-risk MSM who use these sites could be an effective way to encourage them to test and may detect new infections earlier. However, these efforts have primarily been designed to encourage a single test. Mobile/web prevention tools could be an effective way to seamlessly connect with high-risk MSM via the hookup apps/sites they already use and to keep them engaged over time by encouraging them to test regularly and linking them with other prevention resources afterward.

#### HST is underutilized as a testing strategy

Despite HST’s potential, it is not frequently used in community prevention and testing programs due to a number of important challenges [36–38]. Likely the most critical of these is that HST does not provide immediate linkage to care for those who receive “reactive” (or preliminary positive) results, which some suggest may lead to delays in seeking confirmatory testing and care [39]. Modeling studies suggest that, if HST does not provide sufficient linkage to care, its use may actually increase the number of new infections at the population-level because of these delays [40]. In part to address this concern, OraSure® maintains a 24-h, toll-free helpline that HST users can call for counseling, test instructions/guidance, and HIV care referrals. However, this approach relies on consumers to “reach out” for these services, and existing evidence suggests that they rarely do: estimates from OraSure have suggested that few calls have been made to the helpline given the number of tests sold to date and that < 5% of calls were related to post-test counseling or HIV diagnosis/treatment needs [41]. These estimates appear to support concerns that many HST users are not being connected with vital follow-up and referral services after testing, including confirmatory testing/care. It also highlights the need to explore more “active” methods of engaging with HST users after testing to link those who may be positive with HIV care. Conducting more “active” follow-up after HST may also be useful for those who test negative, since it could connect these men with counseling and referrals for other critical prevention services (e.g., STI testing, pre-exposure prophylaxis [PrEP] consultation/care). It could also increase the likelihood of more regular testing, since these contacts could serve as reminders for future testing and could explicitly encourage testing at regular intervals in the future.

Another factor that likely hinders the adoption of HST in prevention programs is the lack of knowledge about its effects among high-risk groups in the real world. For example, some suggest that HST users may feel greater distress/anxiety and less social support when testing alone [42, 43]. Others also cite concerns about “risk compensation” or increases in sexual risk behavior after HST users test negative [44, 45]. HST use could also produce positive effects, however, such as increased empowerment over one’s health or a greater sense of confidence and well-being. Overall, these studies suggest that exploring more active approaches for providing follow-up counseling and linkage/referral after HST could help inform an approach that expands testing and also efficiently connects high-risk MSM with care or prevention services. Examining how these approaches affect testing behavior, receipt of follow-up services, and the emotional/behavioral well-being of users is also critical.

#### The eTest system

**Fig. 1:**
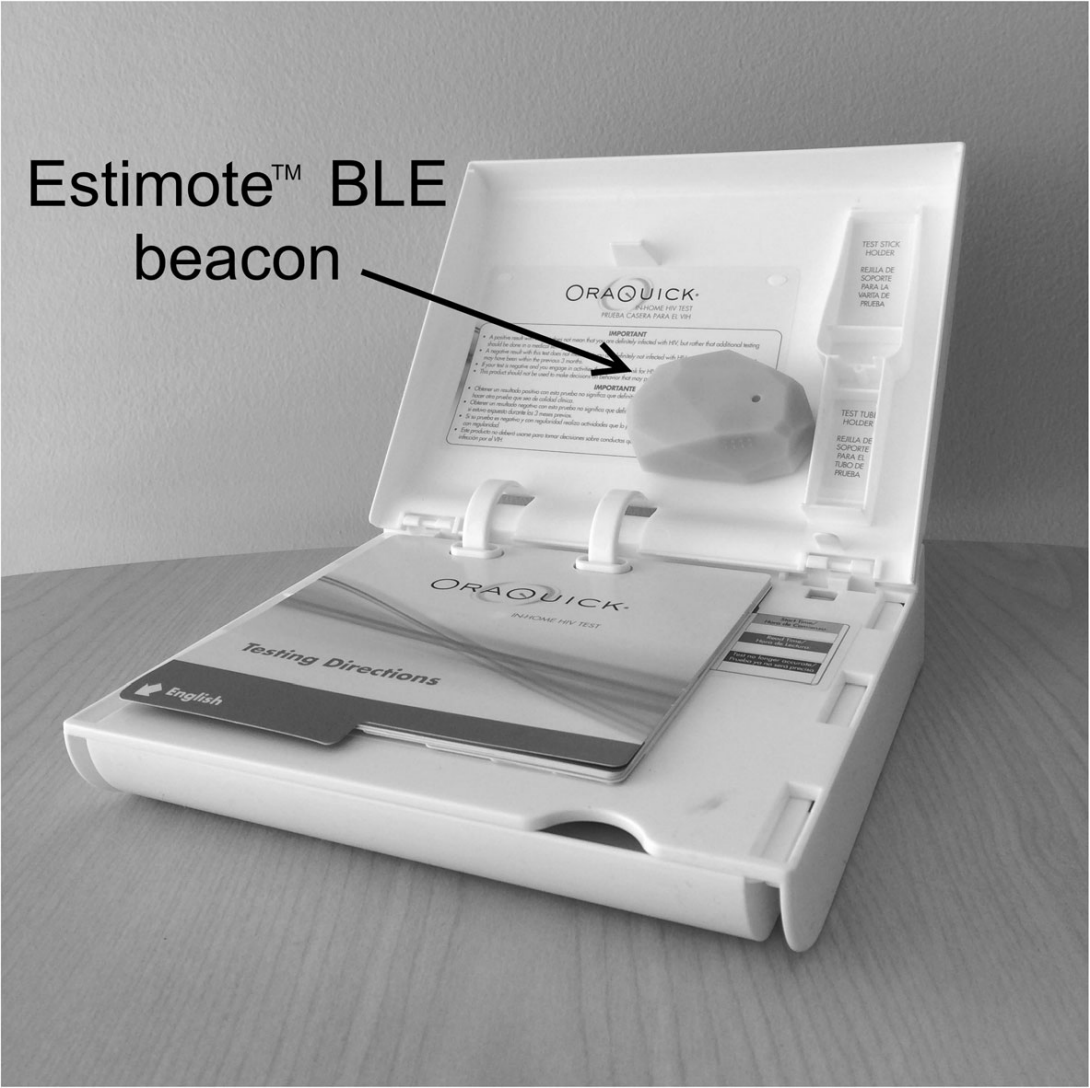
HST kit equipped with beacon

We created a system called eTest that detects when users open HST kits remotely in real time, allowing us to conduct follow-up counseling and referral with these users over the phone. The system, described in detail elsewhere [46], uses a native smartphone application (iOS, Android) installed on users’ devices and Bluetooth low-energy (BLE) beacons placed inside each HST test kit (see Fig. 1). These devices relay a signal through the users’ smartphone to a database when it is activated with the opening of a test kit and is nearby the user’s smartphone. Once a beacon-equipped HST kit is opened, the eTest system notifies researchers that a user may have initiated HST, prompting counselors to call these users to provide post-test counseling and referrals. In a smaller 7-month pilot study (*N* = 65) [47], all participants (100%) in the eTest and standard HST groups reported HIV testing at least once, compared with 72% of controls. More of those in the HST groups also tested two or more times during this 7-month period, aligning with recommendations from the Centers for Disease Control, than those in the control group (79% vs. 41%). Finally, participants in the eTest group were also significantly more likely than controls to receive certain HIV prevention services, like prevention supplies (e.g., condoms and lube) and PrEP referrals. However, we did not find differences in testing for sexually-transmitted infections or initiating PrEP across the conditions. Still, these results suggest that delivering HST kits to high-risk MSM at regular intervals could increase HIV testing rates and encourage more regular testing. Moreover, they also suggest that the eTest system warrants further testing, and specifically, exploring whether providing active post-test referrals alongside HST might also connect high-risk men with some other important services that encourage prevention behaviors.

### Objectives {7}

The proposed research is a limited-interaction, longitudinal randomized controlled trial conducted primarily over the internet. The study tests whether the eTest system and providing home delivery of HST at regular, 3-month intervals increases rates of any and repeat HIV testing, use of other prevention services (e.g., STI testing, HIV risk reduction counseling), and PrEP linkage/initiation compared with “standard” HST and reminders for clinic-based testing in high-risk MSM. Specifically, we will test whether the eTest system results in higher rates of (1) initial and follow-up HIV testing and (2) receipt of additional prevention services (e.g., STI testing, risk reduction counseling, safer sex supplies) compared with standard HST and reminders for clinic-based testing among MSM. We will also test whether providing focused information/counseling about PrEP during eTest contacts results in more participants (3) consulting with medical providers about PrEP and (4) initiating PrEP. Finally, we will also assess the cost-effectiveness of the eTest system for improving rates of HIV testing compared with clinic-based testing alone, exploring whether the eTest system (and HST in general) can be cost-effective under various scenarios (e.g., testing intervals).

### Trial design {8}

In this study, participants recruited from various websites (e.g., social media, gay-oriented sites) were enrolled and oriented to the study online and were randomized 1:1 to a single study condition for the 12-month trial. This design is intended to test the superiority of HST with phone counseling versus passive HST and clinic-based testing alone.

## Methods: participants, interventions, and outcomes

### Study setting {9}

Participants will be recruited from several major US cities intended to provide both broad geographic representation and oversample the South. These cities included Boston, MA, Providence, RI, Los Angeles, CA, New Orleans, LA, Baton Rouge, LA, Shreveport, LA, Jackson, MS, Miami, FL, Orlando, FL, Jacksonville, FL, and Tallahassee, FL. These areas have some of the highest rates of MSM living with undiagnosed HIV [48] in the USA.

### Eligibility criteria {10}

Eligible participants will be (1) males who were assigned male sex at birth, who are (2) not currently on PrEP, and (3) who report any of the following in the past 6 months: (a) anal sex without condoms outside of a monogamous partnership with a recently tested HIV-negative male, (b) an STI diagnosis, or (c) an ongoing sexual partnership with an HIV-positive male. Risk criteria were chosen to align with The Department of Health and Human Services PrEP criteria [49] to focus on recruiting those at highest risk for HIV and are optimal candidates for PrEP. Eligible participants will also (4) have not tested for HIV in the last 12 months, (5) have a stable residence in one of the site metros where they can securely receive packages, and (6) use an iOS/Android smartphone with a data plan or home Wi-Fi, and will be (7) fluent in either English or Spanish. Phone counseling interventions will be conducted by Bachelor’s degree -holding staff members who are certified HIV test counselors in Rhode Island.

### Who will take informed consent? {26a}

Participants will provide informed consent online as part of an online study “onboarding” process. Informed consent information will be provided in written, audio, and video formats, in lay language. Important concepts will be highlighted via bulleted text, highlighted text, and/or captions. A short, two-question “quiz” will assess whether participants understand key consent information.

After providing online consent, study staff will also contact participants by phone to collect further contact information, affirm their commitment to enroll in the study, and ensure their understanding of consent information. Based on these conversations, if staff have reason to believe that a participant is not aware of study requirements or may be unable to provide informed consent, they will notify the participant that they will not be enrolled.

### Additional consent provisions for collection and use of participant data and biological specimens {26b}

Participants will be asked to provide consent for broad use of their online survey and medical service use data, to facilitate secondary data analyses.

## Interventions

### Explanation for the choice of comparators {6b}

We elected to compare real-time, “active” outreach for phone counseling after HST versus “passive” HST given evidence from our previous research that reaching out to MSM and HST may be a good opportunity to connect them with other HIV prevention services (e.g., PrEP). We also elected to include a control group that involved sending text message reminders for participants to seek clinic-based testing to provide a comparison of these HST methods with a low-cost intervention that resembles the standard approach to increasing testing.

### Intervention description {11a}

For control group participants, text messages will be sent once every 3 months to remind them to get tested for HIV at a local clinic. These messages will contain links to a site that provides a concise list of clinics in the participant’s area that provide free HIV testing services and their locations, phone numbers, and hours. For standard HST participants, staff will send an HST kit to their confirmed shipping addresses every 3 months. These participants will receive no phone-based follow-up, but can use OraSure’s provided 1-800 number for questions or needs. For eTest participants, HST kits will also be sent to participants’ confirmed shipping addresses every 3 months. However, each test kit will be fit with an Estimote™ Bluetooth beacon. These beacons automatically detect when each kit is opened and relay this information to a central study database, which triggers an email notification sent to counselors. Within 24 h of opening the kit, an HIV test counselor will call eTest participants to conduct post-test counseling and refer them to other needed services, including PrEP.

### Criteria for discontinuing or modifying allocated interventions {11b}

The interventions will not be modified for any reason.

### Strategies to improve adherence to interventions {11c}

All participants will be instructed to keep the app downloaded as much as possible during their time in the study. Since the app’s database allows us to track when users uninstall it, we will contact participants who delete the app by phone and email to inquire about their interest in continuing with the study. We will also track data on app uninstalls (e.g., reasons for uninstalling and/or withdrawing) to continue examining the usability and burden of the app. eTest participants who change their smartphones during the study period (due to an upgrade, change in service provider, or the phone being lost or stolen) will be able to re-download and login to the eTest app from app stores appropriate to their operating system (iOS, Android).

To avoid influencing the study results, participants in HST conditions (eTest and standard HST) will not be given explicit instructions about whether or not to use the tests sent to them. Participants will be informed that HST kits will be sent to them 1 week, 3 months, 6 months, and 9 months after initially signing up and that they can choose to take these tests or not.

### Relevant concomitant care permitted or prohibited during the trial {11d}

Participants are encouraged to seek PrEP care as part of the study’s intervention. As a result, those on PrEP during screening will be excluded. All other types of care are permitted.

### Provisions for post-trial care {30}

Referrals to local, LGBT-friendly agencies for substance abuse treatment, mental health treatment, and primary care will be provided continuously throughout the trial. Lists of these clinics and services will be generated by research staff, verified, updated as needed throughout the study, and will be provided to participants through the app, and in every test kit package sent during the study.

Participants who test positive with HST (those in either the standard or eTest conditions) will be assured that an initially reactive result is not a confirmed positive result. Counselors will then use three-way calling to assist participants in scheduling an appointment for confirmatory testing at specific designated clinics in each city in which the investigators have existing relationships. These clinics/centers have standard procedures for providing newly diagnosed patients ongoing HIV care. Test counselors will conduct follow-up calls after each participant’s scheduled appointments to ensure that they receive confirmatory testing. Participants with reactive test results will also be screened for suicidality during these calls, and if necessary, intervention will be provided according to National Suicide Prevention Lifeline procedures.

Due to funding limitations, there are currently no plans to continue providing HST to participants after the study has completed.

### Outcomes {12}

Primary outcomes will be the proportion of participants in each group who (1) tested for HIV at any point over the 12-month study, (2) tested within each 3-month interval, and (3) were tested at least once during the CDC-recommended intervals of at least once every 6 months over the year-long study. (4) The proportion of participants who consulted with a physician about PrEP, (5) received a PrEP prescription, (6) received any STI testing during the study period, and (7) received STI testing at least once during each 6-month period, aligning with the CDC-recommended interval of testing for STIs at least once every 6 months. These outcomes will be assessed via self-report in online follow-up surveys collected at 1, 4, 7, 10, and 12 months post-enrollment. Self-report data on clinic-based HIV testing, STI testing, and PrEP uptake will be verified by requesting medical records data from the clinics in which participants reported receiving these services.

### Participant timeline {13}

**Fig. 2:**
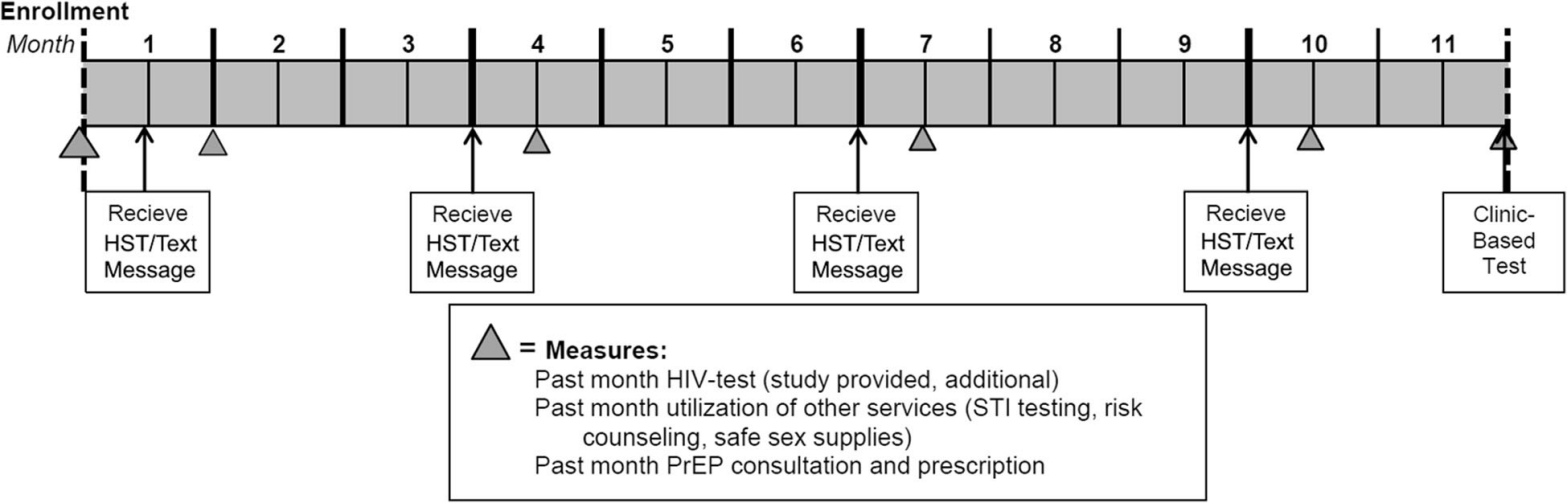
Participant timeline on the study

**Fig. 3:**
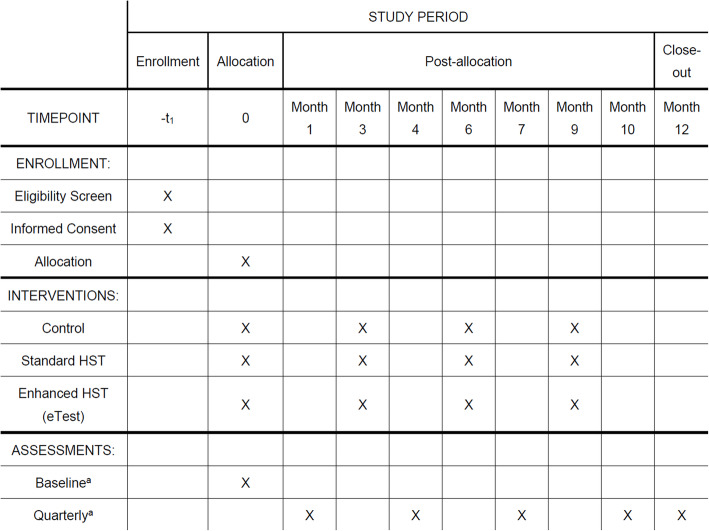
SPIRIT timeline on the study. See above the “Outcomes {12}” section for a full list of primary study outcomes assessed at all assessment timepoints

Participants will be enrolled and randomized on a rolling basis, beginning in January 15, 2019, until January 15, 2022. Once enrolled, participants will receive their assigned study intervention (HST with follow-up, HST alone, or text message reminders) at 1 month, 4 months, 7 months, and 10 months after baseline. They will complete online follow-up questionnaires at baseline, 3 months, 6 months, 9 months, and 12 months. These online follow-ups will collect data on the study’s key outcomes. At 12 months, participants will be asked to present to a local clinic to provide a blood sample for HIV testing for a bonus payment of $50. This procedure will ensure that all participants have at least one HIV test throughout the study period. As this is a “limited interaction” study in which our goal was to explore how these interventions might fare when implemented in real-world scenarios, there were no in-person visits. See Figs. 2 and 3 for study timelines.

### Sample size {14}

We used past studies [50, 51] and GLIMMPSE software [52] to determine the sample sizes necessary to detect significant effects using each of the proposed analysis models in section 20a. In our pilot data [47], differences in most outcomes (both any and regular HIV testing, STI testing, and PrEP uptake) across the HST and control conditions were very large, while differences across standard HST and eTest conditions were small (*OR* = 1.5). Given our interest in whether eTest improves outcomes relative to standard HST, we therefore estimated the sample size necessary to detect small effects across all models. The sample size needed to detect an effect of this size in a logistic regression model (models 1, 3, and 4), assuming *α* = 0.05 and an observed power of .80, was 845. In a mixed-effects regression model testing between-group differences in HIV testing within each of four quarterly assessments using the same assumptions and within-persons correlations drawn from our pilot study (*ρ* = 0.2), the sample size required to detect small effect sizes between groups was 240. As such, we selected a total target sample size of 900 to align with the largest of these sample size estimates, plus some over enrollment to compensate for missed responses and attrition.

### Recruitment {15}

All participants will be recruited via geographically focused advertisements placed on social media (e.g., Facebook, Instagram, Reddit), general search (e.g., Google), and gay-oriented websites and apps (e.g., Grindr, Jack’d, Scruff). These advertisements will appear to those who log in within a specified radius of each identified metro area. One important goal of this trial was to recruit substantial portions of AA and H/L MSM, due to their higher risk for HIV infection (40% AA, 35% H/L). To achieve this, we will use specific ad content that depicts AA and H/L individuals and recruit in cities with large populations of AA and H/L MSM.

## Assignment of interventions: allocation

### Sequence generation {16a}

The allocation sequence was determined using computer-generated random numbers. Groups were not stratified by any factors. Study randomizations are simple; no blocking is used in the randomization scheme.

### Concealment mechanism {16b}

A web application through which participants enroll in the study will automatically assign participants to study conditions without interacting with study staff in any way.

### Implementation {16c}

The web application will generate the allocation sequence, enroll participants, and assign them to intervention conditions. The allocation sequence was generated before the study began.

## Assignment of interventions: blinding

### Who will be blinded {17a}

Neither participants nor research staff in charge of corresponding with participants will be blinded to participants’ study condition. However, the risk of bias from these study staff members is low, because follow-up surveys are not administered by research staff. A study database automatically sends emails to participants in order to facilitate completion. The primary data analyst will be blinded to study condition for between-group analyses of primary study outcomes.

### Procedure for unblinding if needed {17b}

Due to the nature of the interventions, there will be no blinding of participants nor research staff.

## Data collection and management

### Plans for assessment and collection of outcomes {18a}

We will use online surveys to collect all self-report data. These online surveys will be collected at baseline, 1 month, 4 months, 7 months, 10 months, and 12 months. These follow-up intervals were selected in order to avoid priming responses, given that test kits and reminders will be sent at baseline, 3 months, 6 months, and 9 months. On each semi-quarterly follow-up survey’s due date, the study database will automatically send participants an email with language-specific instructions and links to the surveys. Participants will be asked to complete these within 2 days of being sent. Repeated follow-up survey submissions will be discouraged by notifying participants they have already completed the assigned survey if they click the email survey link.

Follow-up surveys will assess HIV testing since the last study assessment, including whether they tested, how/where they tested, what their results were, whether each test was associated with PrEP care, or reasons for not testing. This approach will allow us to track contamination across conditions or the extent to which those assigned to the control condition used a HST or those inHST conditions tested at a clinic. Questionnaires will also assess whether participants were referred for additional prevention services (e.g., STI testing, HIV risk reduction counseling) and whether they received these services since the last survey. Items will also assess whether participants consulted with a medical provider about PrEP in the last month, and if so, their provider’s information and whether they were prescribed PrEP.

We will also review clinical data for each participant to compare with self-report data on HIV testing, STI testing, and PrEP uptake. We will obtain signed HIPAA releases for each service participants reported using, in order to allow these clinics to release relevant records to us in order to verify these services. Data on verified service use will be compared across conditions to explore potential differences. While we anticipate that this data will be incomplete (especially given that some may elect to test at certain sites anonymously), we believe that collecting as much corroborating data as possible will serve as an important compliment to self-report data.

At the end of the 12-month study period, all participants in all conditions will also be asked to present to designated clinics for in-person HIV testing in exchange for a bonus payment ($50). Encouraging participants in this way will help ensure that we have at least one accurate HIV test result for each (since some may elect not to test at all during the study period, regardless of condition). Results will allow us to more confidently estimate the number of new infections that were successfully detected or missed with HST versus clinic-based testing reminders.

### Plans to promote participant retention and complete follow-up {18b}

For participants who fail to complete their online follow-up surveys the day they are assigned, reminder emails will be sent every day for 5 days after the due date. If participants have not completed the survey within 5 days of their due date, research staff will contact participants by phone, text message, and email to encourage adherence. For all participants, those who fail to complete two consecutive quarterly assessments will be considered to have been withdrawn from the study and will not contacted further. Quarterly surveys will also inquire about any changes in participants’ contact information each month.

### Data management {19}

Data will be collected using an online survey platform and will be continuously transferred with each participant submission to a central study database using a web service. Prior to performing analyses, we will conduct range checks to ensure the plausibility of values.

### Confidentiality {27}

Before beginning their work on the study, all members of the research staff will receive thorough training in procedures designed to maintain data security. While collecting and storing participants’ personal information using web-connected databases is unavoidable due to the nature of the study, a number of steps will be used to help secure and safeguard this data throughout the project. All elements of the proposed system (web application, mobile application, database) will be hosted on secure servers that enforce a strict set of security and authentication rules. For all elements containing sensitive or protected information, additional security measures will be applied, such as two-step verification (a password plus registered device), VPN-only access, and IP-specific firewall rules. Access to participant information will be restricted to essential research staff and only after two-step verification. 

Since participants will complete quarterly questionnaires online, and some questions asked will be sensitive, they will be specifically instructed to complete these in a private location when possible. Emails that are automatically sent to participants to remind them to complete the surveys will also remind them to complete assessments in private. Reminder emails will also contain no identifying information (other than their email address) or references to HIV testing or other sensitive topics.

All participants will have access to information about HIV and other sexually transmitted infections, testing, prevention, and referral information via the eTest app. As such, they will be instructed to use their phone’s lock screen to prevent unauthorized access. They will also be instructed to access this information only in private locations. Push notifications and text messages will be worded as innocuously as possible (e.g., “Looks like you opened your test!”). Participants will be encouraged to use their devices’ native security settings (e.g., enabling a lock screen with code in order to access the phone) while they are in the study.

If participants have been assigned to either the standard HST or eTest conditions, OraSure OraQuick® HST kits will be sent to the verified physical addresses of participants throughout the study. To safeguard participant confidentiality, these packages will be sent in discrete packaging. An information card will also provide participants with tips for ensuring their privacy while taking the test at home and for disposing of the test collection swab. OraSure provides an envelope container that participants can use to confidentially dispose of the test collection swab after the test is complete.

When conducting follow-up phone calls, counselors will first ensure that participants have adequate privacy to discuss the test over the phone, and if not, calls will be re-scheduled. Data from these calls will be manually entered by counselors into the study’s central, password-protected database. Occasionally, counseling phone calls will also be digitally recorded for training, supervision, and fidelity purposes. These recording files will be password-protected and stored on the study’s secure servers, in locations separate from participants’ identifying data. Digital audio recordings collected for supervision purposes will be deleted immediately after supervision meetings have occurred. Those used for training and fidelity will be stripped of any identifying information that may have been recorded and deleted after they have been used.

Once the study is completed, we will create a compiled dataset with all collected study data, and remove any identifying information. Once this de-identified, archival dataset is created, we will destroy any original participant identifying information.

### Plans for collection, laboratory evaluation, and storage of biological specimens for genetic or molecular analysis in this trial/future use {33}

Not applicable.

## Statistical methods

### Statistical methods for primary and secondary outcomes {20a}

To test whether rates of any testing differed across the three study arms, we will use factorial logistic regression. Dummy-coded variables reflecting study condition will serve as the focal predictors. To explore the effects of study condition on regular HIV testing, we will use multilevel models for repeated, binary outcomes, with a dummy variable reflecting whether participants tested during each 3-month study period as the focal outcome. A logit link function and independent correlation structure will be specified, with time and study condition as a focal predictor to test whether the odds of testing differ across time period and condition. For all models of HIV testing outcomes, a covariate reflecting whether a given participant had initiated PrEP (and when, for longitudinal models) will be added to each model, since national guidelines require these individuals to be tested for HIV quarterly as a part of ongoing PrEP care. Doing so will allow us to estimate the effects of study arm on HIV testing among those who did not initiate PrEP. To test whether the conditions differed in terms of the number of participants who sought consultation about PrEP or ultimately initiated PrEP, we will estimate factorial logistic regression with a dummy-coded indicator for study condition as a focal predictor. Finally, to test whether the study conditions differed in terms of the number of participants who received STI testing at any point during the study period, we will estimate logistic regression models, with dummy-coded variables for study condition serving as focal predictors.

### Interim analyses {21b}

No interim analyses will be performed. The trial will be terminated when the target sample size is reached.

### Methods for additional analyses (e.g., subgroup analyses) {20b}

No additional subgroup analyses are planned.

### Methods in analysis to handle protocol non-adherence and any statistical methods to handle missing data {20c}

Data from those who drop out or withdraw from the study will be used in these analyses in intent-to-treat (ITT) fashion [53]. Depending on the degree of missing assessments, we will use multiple imputation for these values [54].

### Plans to give access to the full protocol, participant level-data and statistical code {31c}

After all participants have finished completing study procedures, a de-identified, archival dataset will be created. Once created, this dataset will be uploaded into the National Institute of Mental Health’s data archive in accordance with institute policy. The dataset will be provided to investigators upon request after an embargo period of 2 years. Analysis scripts associated with all publications will also be made available upon request.

## Oversight and monitoring

### Composition of the coordinating center and trial steering committee {5d}

The study’s scientific team (T. Wray, P. Chan, J. Klausner, L. Mena, and J. Brock) will serve as the primary steering committee and jointly make all decisions about the course and conduct of the study. All members are physicians, most with training in infectious diseases, with the exception of Dr. Wray, who is a clinical psychologist. Formal meetings of this steering committee will occur at least monthly, with informal communication occurring weekly or more often.

### Composition of the data monitoring committee, its role and reporting structure {21a}

As this trial tests several interventions known to improve HIV testing rates, multiple ethics review committees (e.g., sponsor, Institutional Review Boards at each of the investigators’ home institutions) have determined that no data monitoring committee was necessary for this trial. That is, since each of the interventions being studied (HST, text message reminders) only improve upon the standard of care, the risk was insufficient to warrant close, independent monitoring of the study results.

### Adverse event reporting and harms {22}

Adverse events and other unintended effects are collected from research participants systematically in online surveys. There are no adverse events expected, as this is a minimal risk study. However, if an adverse event is reported, the study’s contact PI (Dr. Wray) will complete an adverse events form and report the event to the Brown University Institutional Review Board (IRB) within 24 h. The PI will also report adverse events in writing to the study’s sponsor. The PI will then gather any information needed to investigate the event and to determine subsequent action and will document and report any subsequent action to the IRB and the sponsor. We will also generate a brief report of adverse events for the study record each year, and we will forward the report to the IRB and the sponsor. We will report all adverse events in trial publications.

### Frequency and plans for auditing trial conduct {23}

No procedures are planned to audit the trial conduct.

### Plans for communicating important protocol amendments to relevant parties (e.g., trial participants, ethical committees) {25}

Amendments to the trial protocol will first be requested from the home institution’s IRB. Once approved, these amendments will then be reflected in the trial protocol, and a revised version will be updated on this study’s trial registration page. Any other information in the registry will also be changed to reflect the amendment. Major changes to the scientific direction of the project will be requested from the study’s sponsor and approved prior to changing the conduct of the trial.

### Dissemination plans {31a}

Results of the primary outcomes identified in this trial will be published in relevant scholarly journals. Investigators will also present these results at relevant national conferences. Results will also be uploaded into the trial registry after they are available. We will also upload a final, de-identified archival dataset to the National Institute on Mental Health’s data archive. This dataset will be made available to requesting investigators after an embargo period of 2 years.

## Discussion

We believe one of HST’s key strengths is its potential to reach high-risk MSM who do not otherwise engage with traditional, “brick-and-mortar” services [22–24]. It may also be a particularly effective strategy for engaging many AA and H/L MSM, who may be reluctant to use traditional services due to medical mistrust and fear of stigma [26–28]. We designed this study as a practical, “limited interaction” trial in order to approximate what a program that provides regular, home delivery of HST might look like if it were implemented in actual communities, focusing on “harder to reach” MSM who may be better served with HST. However, we also balanced these goals with the need to collect thorough and valid data on the effects of programs like these over time. For these reasons, we elected to recruit and enroll participants entirely online and use infrequent online surveys as follow-up, in order to avoid the barriers involved in face-to-face appointments. Although we realize that this will likely present difficulties in terms of ensuring the validity of data collected, several steps involved in our procedures were intended to help address this. First, multiple online signups from the same IP or device will be blocked, and participants must verify their email addresses when registering, as well as other contact information via a call with staff after enrollment. Second, in this brief enrollment call, research staff will ensure each participants’ understanding of the study procedures and commitment to completing the study procedures before considering participants fully enrolled. Third, participants who report having received HIV prevention or sexual health services at a clinic in each follow-up survey (e.g., HIV testing, STI testing, risk reduction counseling, PrEP consultation, PrEP initiation) will be asked to submit a signed release within that same survey requesting that these clinics release data from the medical records of these patients to our research team. Staff will then send this release to each clinic and request that these data be shared with our research team. These data will be tracked in a central study database and used to validate participants’ self-report. Together, we believe the described procedures balance being as “hands off” as possible with conducting regular follow-ups and validation steps that allow us to understand the effects of these programs thoroughly and accurately.

The results of this study will ultimately help us understand how much regular, home delivery of HST might improve rates of HIV testing and promote regular testing, relative to a lower-cost strategy of simply reminding MSM to get tested at local clinics at regular intervals. It will also help us understand whether providing real-time counseling and referrals over the phone after HST use helps connect more MSM with needed HIV prevention services like risk reduction counseling, STI testing, and PrEP, when compared to passive HST or clinic-based testing alone. If this research supports the use of eTest to help connect MSM with prevention services, future research should explore strategies for implementing similar programs in highly affected communities in the USA. Future research could also focus on improving methods for identifying those at highest risk within various communities, engaging them in similar HST home delivery programs, determining optimal HST intervals for detecting new cases in these individuals, and changing community stakeholder attitudes about HST.

## Trial status

Protocol version number 1 (created January, 2019). Recruitment began on January 15, 2019, and is slated to conclude on January 15, 2022.

## Abbreviations

MSM: Men who have sex with men
HIV: Human immunodeficiency virus
STI: Sexually transmitted infection
PrEP: Pre-exposure prophylaxis
HST: HIV self-testing
